# “It’s business as usual”: adolescents perspectives on the ban of alcohol sachets towards reduction in under age alcohol use in Malawi

**DOI:** 10.1186/s13011-020-00280-8

**Published:** 2020-06-03

**Authors:** Sangwani Salimu, Alinane Linda Nyondo-Mipando

**Affiliations:** 1 Department of Public Health, School of Public Health and Family Medicine, College of Medicine, Blantyre, Malawi; 2 Malawi-Liverpool-Wellcome Trust Clinical Research Programme, College of Medicine, University of Malawi, Blantyre, Malawi; 3 Department of Health Systems and Policy, School of Public Health and Family Medicine, College of Medicine, Private Bag 360, Blantyre, Malawi

**Keywords:** Adolescents, Alcohol policy, Alcohol sachets, Snow ball sampling, Availability, Accessibility, Role modelling

## Abstract

**Background:**

Alcohol contributes to poor health, social and economic outcomes among adolescents. In Malawi, alcohol consumption among young people significantly increased after the introduction of alcohol sachets. A government ban on the sale of alcohol sachets affected in 2012 aimed to reduce prevalence of alcohol among users. We explored adolescents perceptions regarding the effectiveness of the ban towards reducing alcohol consumption among the under aged in the country.

**Methods:**

Using a descriptive phenomenological school-based approach, we recruited 44 school-going adolescents, 15–17 year olds using snow ball sampling and conducted 12 individual semi-structured interviews and four group discussions differentiated by sex. We sought a waiver from College of Medicine Ethics Committee (COMREC) to obtain verbal consent from adolescents. All interviews and discussions were digitally recorded and simultaneously transcribed and translated verbatim into English. Data management and analysis was done manually using thematic approach.

**Results:**

Aggressive packaging, and marketing tendencies and lack of restrictive measures in Malawi have rendered the ban ineffective through increased affordability and availability to different income population groups and the underage. Results indicate that even though adolescents perceive the ban as a significant step towards reducing under age alcohol use, personality and drinking motives precede any interventions. Adolescents emphasized on strong personality as a significant factor for reduced alcohol intake or abstinence.

**Conclusions:**

We recommend strict alcohol policy and enforcement regarding packaging, pricing, positive role modelling by parents and enhanced adolescent personality development through schools and families.

## Background

Globally, alcohol remains one of the main risk factors contributing to the burden of disease among young people, while age of initiation in most developing countries has dropped [1]. The average alcohol consumption among adolescents in sub-Saharan Africa (SSA) remains below the global or African region [1]. Young alcohol users in developing countries are likely to be resident in urban areas, rising in affluence and identify with a western as opposed to a traditional cultural identity [2]. By 2016, 55.1% of current adolescent users in Africa engaged in heavy episodic drinking (HED) [1]. A systematic study on substance abuse among adolescents in SSA reports alcohol prevalence rates at 40.8%, second to tobacco at 45.6% [1]. In general, the youth in developing countries remain vulnerable to alcohol use [3].

Factors related to under aged drinking in SSA include peer pressure, role modelling by parents and family members, poor social and coping skills, positive expectancies from drinking and increased alcohol availability [4, 5]. Literature attributes the increase in alcohol use in developing countries to the expansion of the global alcohol market, packaging, pricing and aggressive marketing techniques [6]. Alcohol intake among adolescents is associated with lower academic achievement, school drop-out, risky sexual behavior, poor health outcomes and a high likelihood of alcohol dependency in adulthood [4]. Additionally, alcohol use among early adolescents and youth commonly co-occurs with other substance use and problem behaviors like tobacco and, other substance use, poor academic performance, school absenteeism, risky sexual behaviors and drink driving [5].

In general, there is a paucity of data on alcohol consumption in Malawi, particularly among the underage. However, it is well documented that emerging trends are in sync with global patterns such as increased binge drinking among the youth, diminishing gender differences and decreasing age of initiation [5, 7]. A study in Malawi reports decreased age of initiation to 10 [5] while a study in Nigeria reports 38% of adolescents had their first drink between the ages 14 and 17 as compared to 33% in the United States initiating at 15 [8]. Contrary developments are reported in Nordic countries (Denmark, Finland, Iceland, Norway, Sweden and Greenland) which report safer drinking patterns, decreased prevalence and increasing age of initiation among the youth [9]. Notably, Nordic countries also report reduced health and economic inequalities, increased supervision of youth by parents, decreased alcohol availability and strict enforcement of alcohol policies [9]. While SSA register high abstinence levels among youth, this is overshadowed by high consumption rates per user [10]. With population age structures characterized by an ever growing youthful base, developing countries have long been targeted for market expansion with affordable alcohol such as liquor sachets [6, 11]. In Malawi, this scenario is exacerbated by weak regulation reaching peak levels during period of alcohol sachets [2, 5]. A national survey conducted in 2012 to inform on alcohol misuse and alcohol policy making reported high abstinence levels and low female consumption in the country [2]. However, the report also confirmed increasing rates of HED among all users [12]. Similarly, in 2019, Uganda banned the sale of alcohol sachets in the country, citing increased use among the youth due to increased affordability [13].

Prevailing trends at the time of the ban of alcohol sachets in Malawi indicated dangerous use of alcohol among youth [14]. Media reports were awash with reports on abuse of alcohol sachets among underage populations as evidenced by obvious intoxication among youth and lack of implementation of alcohol policy [13, 15]. Further, the under aged could be seen drinking in public and teachers reported obvious intoxication among students [5, 12]. To reverse the trend, the government banned the sale of alcohol sachets in 2012. Alcohol manufacturers responded with a court injunction to the ban. A long battle followed between government and manufacturers ending up with a stay on the ban on the 4th of January, 2016 [15, 16].

Prior to 2014, no studies had been conducted on alcohol sachets in Malawi until a qualitative study nested within a national alcohol survey [12] looked at the context and consequences of liquor sachets use among young people [5]. The study reported significant findings: increased alcohol consumption among the youth, decreased age of initiation and a positive association between underage alcohol use and alcohol sachets [5]. Major contributing factors leading to underage liquor use included minimal packaging and pricing as well as lack of restriction in sales. Sachets ranged from 30-100mls while the prices ranged from K10–30, equivalent to 2–8 US cents [5, 17, 18]. Since the ban and Hoel et al., no research has been conducted in the country to explore the effectiveness of the ban towards reducing underage consumption. We bridge this gap by describing the landscape regarding underage alcohol consumption among adolescents in the country.

## Methods

### Design

We employed a descriptive phenomenological school-based qualitative approach to explore adolescents’ perceptions on the effectiveness of the ban on alcohol sachets towards reducing underage consumption. In total, we conducted 12 in-depth interviews (IDIs) and four heterogeneous focus group discussions (FGDs) with 44 adolescents. Considering the limited sample size, we employed method triangulation in order to enhance data saturation and validity of findings through exploration of findings from different perspectives [19].

### Research setting

The study was conducted at two secondary schools in Ndirande, Blantyre. There are three public secondary schools in the township, one of which was used for pilot interviews. The township was selected on two fronts: it is the most densely populated and impoverished township in Blantyre [20] with half of its population being youth under 25 [21]. Before deciding on the study center, the researcher physically visited three most populated townships and surrounding neighborhoods in the city and checked the density of alcohol selling points. This included conducting informal interviews with random residents to ensure chances of finding the phenomena under study as well as increase the possibility of getting required information. The two schools were selected based on their closeness to Ndirande market itself where alcohol is readily available. Further, the day school is at the center of the township and the boarding school is within a walking distance and is also the only secondary public boarding facility in the area.

### Recruitment and selection

Participants were drawn using snow ball sampling and included adolescents 16–17 years old. We purposively selected the head boy and head girl in each school to identify seed participants and, further, we used the seed participants to give us the name of at least one more potential participant and in turn, the potential participants also gave in names of others through snow ball sampling. We choose the head boy and head girl because of their roles as informed persons on individual student behavior and also because they might be in a better position to recruit specified characteristics as opposed to using official channels since adolescent alcohol users might be an unidentifiable group and untraceable through administrative records. The head boy and head girl were asked to draw seed participants using the following criteria:

- Boy or girl between the ages of 15–17
- Willingness to participate

Thereafter, the seed participants were asked to recruit other participants following the same criteria and were also asked to be as diverse as possible in terms of sex, age and class.

The following were used as exclusion criteria:

- Unwillingness to participate
- Adolescents admitting to drinking alcohol 8 hrs prior to the interview
- Adolescents showing any signs of alcohol intoxication such as smelling of alcohol, difficulties in concentrating, slurred, or incomprehensible speech, confusion or failing to coordinate activities such as walking.

The head boy and head girl were sensitized on the importance of maintaining neutrality when approaching and recruiting seed participants to avoid scaring them. The researcher reminded the student leaders that some societies in the country are predominantly conservative and therefore do not condone adolescent alcohol use. Additionally, alcohol use by secondary school students is punishable by law. Accordingly, the head boy and head girl were advised to politely ask potential participants if they were current, former or have ever used alcohol (or alcohol sachets) and recruit without probing on frequency, amounts or anything related to adolescents’ drinking behavior. To avoid prejudicing the researcher and to limit researcher bias, we advised the head boy and head girl not to disclose this information to both researchers (the Principal Investigator-PI and Research Assistant- RA). To further ensure diversity of participants and maintain some degree of control over sampled population [22], we asked the head boy and head girl to recruit both users and non- users as seed participants. Enrolling the desirable characteristics enhanced the probability of getting more in depth findings [22].

Once potential participants were introduced to the research pair, the PI further screened participants by asking the following question: are you able to form an opinion regarding alcohol based on any of the following: your experiences, feelings, knowledge, interactions with peers or family or any other people, religious, health or social beliefs? Before posing this question, we highlighted to students that we were interested in a closed answer (yes or no) and therefore did not solicit follow up responses. The aim was to include students with high and low traits of self-efficacy so as to balance our sample for more representative findings. We therefore deliberately included participants who hesitatingly responded as well as those who responded immediately.

Recruiting stopped upon reaching saturation point. Snowball sampling was used because of its ability to enroll hard-to-reach and hidden populations [23] since under age alcohol use is illegal [24] and a punishable offence in primary and secondary school in the country. During interviews and discussions, the PI was alert and still looked out for signs of drunkenness or in toxicity among participants. Where a participant would have displayed signs of either despite having successfully undergone the screening stage, protocol specified that we immediately stop the interview and delete data collected so far. Three screened participants were excluded because two refused to discuss the subject of alcohol while the other one preferred to be interviewed on a Sunday which was contrary to protocol specifications that interviews and discussions would be conducted on weekdays and within school premises.

### Interviews and discussions

A semi-structured interview guide was developed based on the scoping literature review and was piloted at a secondary school within the same township. Pilot interviews were used to refine tools and was included in analysis. Prior to data collection, the PI trained the RA on expected conduct in the study. All interviews were conducted by the PI while the RA assisted with coordination of discussions and interviews. Because RA was also in contact with participants, he was trained on the importance of maintaining an impartial stand to avoid influencing participants. Tool development was guided by study objectives and the psychosocial factors on student drinking as used by the National Institute on Alcohol Abuse and Alcoholism (NIAAA) [25]. The framework looks at the individuals’ drinking behaviour in the context of combined environmental, cognitive and biological influences [25]. Specifically, it looks at cognitive development and the relationship between certain personality traits and drinking behaviour and relates that to individual expectations from drinking [11]. The topic guide focused on why adolescents drink, knowledge and perception on the effectiveness of the ban on alcohol sachets towards reducing underage consumption. The broad questions that guided the interviews were as follows:

1. Explain to me in detail how alcohol sachets affected adolescents’ use of alcohol in Malawi?
2. Explain to me in detail how the ban of alcohol sachets has affected underage alcohol use?

Participants were constantly reminded that all information gathered would only be available to the researchers and that they were at liberty to disclose (or not) their alcohol use status. We conducted 12 IDIs (six per school and three by gender per site) and four FGDs (two at each school, separated by gender). Interviews lasted about 45–50 min while discussions lasted about 1 h and included 8 participants per group. Interviews were conducted at times and places convenient to participants. We employed data triangulation to mitigate researcher bias, add depth to our findings and we ensured data validity by performing member checking after concluding each section during interviews and discussions [26]. We did not conduct any repeat interviews.

### Data analysis

The interviews were digitally recorded and simultaneously transcribed and translated verbatim into English by the PI. The completed transcripts were then checked for accuracy by a trained research assistant and finally, the research supervisor. Data analysis was manually performed using thematic analysis as outlined by Braun and Clarke [27] and commenced during data collection period. After reading and re-reading the transcripts to familiarize herself with the transcripts, with each reading, the PI developed short notes before developing a codebook that was both inductive from the data, and deductive from the study objectives and the psychosocial framework as used by NIAAA. The research supervisor then checked the codes, refined or added where necessary after consultations with the PI and in reference to the transcripts. Both the PI and research supervisor then worked together in open coding one interview and one discussion. During this process, codes were compared and areas where coders differed were discussed until a consensus was reached regarding the most plausible code for that aspect [27]. Thirdly, all transcripts were then coded by the PI following the coding framework and the coding process and outputs were reviewed by the research supervisor. We searched for themes from the codes by collating all similar and recurrent codes under an overarching theme [27]. NIAAA framework (National Institute on Alcohol Abuse and Alcoholism (NIAAA), 2002) tenets provided the overarching themes for the study while sub-themes were realized from the data. We examined each code for further subcategories (Braun & Clarke, 2006). Fourthly, we reviewed the themes and this resulted in maintaining, combining, separating or discarding unsupported themes as deemed necessary [27] and the decision to change was dependent on the richness and breadth of the proposed theme to accommodate other sub themes. In cases where different themes relayed one message, they were grouped to yield the best fit. Fifthly, we maintained an audit trail and constant comparison of data categories against original transcripts in order to increases validity of findings [27, 28].

### Ethical considerations

We obtained permission from the Ministry of Education, Division Office to conduct the study at the two schools. Further approval was obtained from the administration of the chosen schools. Ethical approval to conduct study was obtained from Malawi’s College of Medicine Research and Ethics Committee (COMREC). We obtained a waiver to use verbal consent with participants in order to assure them of their privacy considering they were underage (under 18) [29]. We could not provide any financial incentives due to budget limitations. Further, there was no need for transport refund as students were already in school for lessons. All interviews were arranged on the day of data collection so all students involved would already have been in school. However, we provided snacks and a drink as refreshments after interviews. Consent was obtained prior to commencement of any study activities. To maintain participants’ confidentiality, anonymity and privacy, we conducted all interviews in a private and quiet room at the schools and used numbers instead of names. All audios and transcripts were saved in a password protected computer with access limited only to the PI and research supervisor.

## Results

### Participant characteristics

Of the 44 adolescents, 23 were boys and 21 were girls aged 15–17, with a median age of 16. Of these, 12 were in Form 2, 7 in Form 3 and 15 in Form 4. We enrolled 24 students from the day school while the rest were from the boarding school.

### Understanding reasons behind adolescent alcohol use

Three main themes emerged from the discussion in relation to adolescent perceptions regarding the ban and reduced use among adolescents. These were (a) availability of alcohol, (b), access to alcohol and (c) lack of restrictive measures in alcohol sales.

#### Availability of alcohol

Adolescents argued that ease of contact with alcohol sachets played a significant role towards influencing alcohol intake among the age group through limitation of physical barriers. One participant described availability as follows:

*“Alcohol sachets were sold everywhere…bus stands, in the minibus, in the market, hawkers within the neighborhood, make-shift stalls. Vendors would walk around with a string of sachets.”*

Echoing the participant above, another adolescent added an element of timelessness to the availability of alcohol sachets as follows:

*“You could buy sachets literally anytime…early in the morning minibus touts would be drunk and one would wonder that how they can be drunk so early in the morning. But that was because they could buy them anywhere anytime so it was easy for them to take early in the morning.”*

Another participant argued on recognition of alcohol sachets by young children to say sachets had become part of a norm, hence even young children knew them.

*“My kid sister who is 10 was laughing at a man the other time because he is always drunk, so my sister said he buys sachets on the way to work and on the way home.”*

### Accessibility of alcohol sachets

Affordability was discussed as an aspect of accessibility noting even the underage could buy alcohol sachets with little effort and without having to worry about any financial limitations. As gathered from one FGD, 100 ml of alcohol was sold as cheap as K30 per sachet (less than US$ 0.08). The quotation below comparing the price of food to that of alcohol illustrates:

*“You do not need a lot of money to get alcohol. Even from your pocket money for a day, you can use some to buy alcohol and still manage to buy something like a banana or cassava to eat during lunch time. As for where to buy it, it is available almost at every place where there is a market of some sort.”*

Access was also discussed in the context of affordability with under age being able to buy alcohol sachets out-of-their pockets but not feeling any financial pinch.

*“They can afford to buy anytime without even having to borrow money. Sometimes, there are some things which if the youth have to buy, they have to ask or beg for money from friends or parents- like for girls to do their hair…but with sachets, the youth spent the little they had and would still have some money left for small things to buy and eat.”*

One adolescent expressed the view that ease of access to sachets was tempting to some adolescents due to the low prices as compared to other healthier options:

*“All you needed was K30 and you were set to go. You did not need to save pocket money to afford alcohol sachets. The sachets were so cheap, it was actually less costly to buy them than it was to buy two bananas. Imagine two bananas would be selling at K50 and one sachet would be selling at K30. No wonder teen agers would sometimes buy the sachets than food.”*

Adolescents noted with increased availability and accessibility, underage populations who drink are more likely to drink more.

*“I started with a sip… would steal my dad’s beer. Then sometimes I would buy my own and my dad caught me drinking and shouted and threatened that he would send me to the village to leave with my mum. But it was difficult to stop, I would buy sachets and still drink and now I would drink more because of sachets. I became addicted, now I am learning to stop.”*

*“Sometimes we would meet with friends just to relax…you know when you are young. Then it became a habit. Now I drink every day.”*

### Lack of restrictive measures to alcohol sale to minors

Adolescents argued that since the emergence of sachets, there were no limiting measures applied to the sale of alcohol to minors adding that the removal of barriers increased access:

*“Anyone could buy alcohol at any time, what mattered was that you had money to pay. They didn’t even ask your age or who sent you to buy the sachets. It was business … all the seller wanted is money.”*

An adolescent whose father owns a tuck shop that sold alcohol sachets collaborated the above:

*“Sometimes I sell in the shop. Teen agers come to buy alcohol. We sell it to them without asking anything. For us, what we need is money and they are bringing money by buying the alcohol. That is our source of income, our school fees. We would chase away customers if we had to ask the buyers anything personal like their age or who had sent them to buy the alcohol. It’s business as usual.”*

Further, another adolescent added on the lack of restriction in terms of retail outlets:

*“Any shop can sell without issues of license, it does not matter what type of shop it is … imagine the little shops in Ndirande, a bar, a fast food take away … ..May be neighbourhood police should take part in checking shops. As residents we can agree, maybe then people will stop selling anyhow they wish.”*

*“I think it’s up to you, rules will be there but you can choose to obey or not. So if you still want you will drink, if you don’t want, then you won’t. There are rules, we know you should not drink unless you are 18 or over. But it’s really up to you.”*

Participants also expressed concern over the lack of restrictions to higher risk alcohol sachets. This was in reference to brands with high alcohol percentages arguing for the need of strict selling restrictions.

*“With alcohol volume at 40%, most of the youth cannot even handle that so most would get drunk and everyone would know that these are the effects of sachets from the way they were behaving. May be if they had lowered the volume of alcohol or made it a law that young people cannot buy alcohol with high volume.”*

### Influence of family and peers

Participants also emphasized the role played by family and peers in influencing under age alcohol use. Some users expressed views that they drunk because their fathers also did. As expressed by male adolescent:

“*My father drinks, so why can’t I? I see him all the time, my mother does not like it but he still does. So sometimes I also taste, not a lot but I just taste.”*

Adolescents also mentioned that sometimes they drunk because of influence of friends. One adolescent mentioned that it felt good to drink when in the company of friends, noting this came with social rewards such as acceptance by peers.

*“You want to be seen to be cool … so when someone invites you, ya, you join. You don’t want to be seen like the odd one out, and sometimes it looks like friends are having fun when drinking, so you also want to have fun.*

Participants related alcohol intake to affluence, noting those who drink think it’s modern.

*“When you see the ones who drink, they think they are cool, they drink and sag their trousers and walk around blasting music from their phones …it’s like a new culture, modern culture. You want to be like them so you also drink.”*

### Recent packaging: a step backwards

An important development post the ban on sale of alcohol in sachets relates to new packaging practice. Manufacturers now sell alcohol in bulk (5 l and above) and, vendors buy this and re-pack as per customers’ demand. The new packaging practice renders the alcohol readily available to underage populations through increasing accessibility.

“*The fact that you can buy at very cheap prices doesn’t help. All you need is K50 and you are set to go, just tell them how much you have in your pocket and you will be given the alcohol. They give you in a cup, according to the money you have in your pocket”*

Some participants added that although the ban was a good intervention towards reducing alcohol use among adolescents, abstinence demands willpower and that individuals need to make personal decisions to stop drinking.

*“The decision to drink is personal. If you make up your mind that you want to stop drinking, you will stop irrespective of the fact that the alcohol is found everywhere and at cheap prices.”*

Also important is that the new selling practice offers convenience to users in terms of hiding the product. Adolescents would buy and, put it in a water bottle and could drink in public without attracting any attention: One participant added:

“*You see them drinking from a water bottle and you will not know its alcohol. Because the drink is clear so they just pretend that it is water. I think the other boys can even drink in school. They are able to drink at any time of the day because they can easily hide it.”*

## Discussion

This paper explored adolescents’ perceptions regarding the effectiveness of the ban on alcohol sachets towards reducing alcohol consumption among the underage in Malawi. Our study is conclusive that the ban had potential towards reducing underage consumption but that is undermined by aggressive packaging adopted by alcohol manufacturers and vendors post the ban. We have demonstrated that the new packaging practices renders the alcohol more readily available and affordable than before the ban was implemented.

We report a positive relationship between alcohol availability and underage use. Similarly, ease of convenience due to selling practices is also confirmed in another study in Malawi [5] and a multi- country study on alcohol use in SSA [11]. Further, our study participants also argued on the relationship between adolescent personality and alcohol use due to ease of availability and convenience, arguing weaker personalities were more likely to succumb to drinking. This finding is consistent with multiple studies which report impulsivity and risky behaviour among adolescents as consistent traits among alcohol and substance users [30, 31]. Further, adolescents in the current study reported increased frequency in drinking.

We also highlight increased prevalence of problem drinking among the underage as a result of access to cheap alcohol, particularly, in regard to number of adolescents using alcohol, frequency of use, and increased amounts of alcohol consumed. Similarly, a study in Ghana noted that the prevalence rates of alcohol use among the youth in the area were two times higher than the national average due to ease of availability and access [32]. The Ghanian study adds that the study area is clustered with bars and entertainment places serving alcohol and that these premises lack policy enforcement on underage use, an environment also reported in the current study. Problem drinking is also reported in Tanzania [33] Uganda [34] and Malawi [5] with all studies reporting increased prevalence and decreasing age of alcohol initiation.

Similar to our results, a number of studies have also reported lack of policy restrictive measures and concluded that because of lack of enforcement, some adolescent users were not aware of the existence of such laws and that those who were, noted the lack of enforcement of the same [35, 36]. However, this study also highlights the fact that knowledge of restrictive policies is not directly related to abstinence among adolescents. This may partly explain why adolescents continue to drink spirits years after the sachets ban. Although some adolescents expressed knowledge that they drink for fun, possible contributing factors to their drinking could be explained through their rebellious nature. Similar findings were also reported in a study among adolescents in 40 European countries [37] and Malawi [5] and Tanzania [33].

A recurring theme was that abstinence or reducing alcohol use amongst adolescents is a personal decision that also hinders on self-esteem, with weaker personalities more bent on non-abstinence. Coupled with sensation seeking behaviour, adolescents with poor self-esteem are less likely to abstain than those with stronger personalities. This finding is confirmed in a systematic review of papers on adolescent drinking worldwide [38, 39].

Prior research indicates peer pressure as a significant influence towards adolescent alcohol use [40]. We report adolescents’ use of alcohol in order to feel a sense of belonging. Adolescents reported drinking like friends because it made them feel socially accepted by others. Similarly a study reported adolescents’ peer use as a seal of approval [41]. This finding is consistent with another study that sought to define adolescent peer perceptions on alcohol and its relatedness to alcohol use, noting adolescents with lesser perceived notions of peer pressure drank less [42].

Some scholars indicate a gender sensitive response to parental influence on adolescent alcohol use with girls responding more to maternal use and boys succumbing to paternal use and more especially to peer influence [43, 44]. Another study reports parental use and permissive attitude as enablers to alcohol use among teens [44]. A systematic review on parental modelling and gender response among adolescents alcohol use is inconclusive [44]. In the absence of a conclusive decision on the subject, one clear outstanding finding is the positive association between parental use to adolescent’s own use of alcohol [45]. Arbar et al., 2009 notes alcohol misuse was more common among boys whose parents approved of alcohol use and also among teens whose parents approved use within certain limits [44]. We also report on a positive link between parental drinking and adolescent involvement with alcohol, as presented by adolescent boys. In light of this positive association as reported by this study and those from different contexts, we highlight the need for further studies on dynamics of parental modelling of alcohol and its effects on adolescent use in our settings. Also notable is the finding by multiple studies that where parental involvement is high, it is linked with weaker relations between adolescents and peer influence on alcohol use and related problems [43, 46]. This involvement includes monitoring of adolescents, enhanced communication between parents and their children as well as personal skills to enable the children to cope with peer pressure [45, 46]. The window therefore presents an opportunity to intensify parental influences in order to minimise peer influence on adolescent alcohol use.

### Limitations of the study

The study had a number of limitations. First, the study findings are limited in terms of generalizability because of sample limitations. Our sample was small and was also limited to school going adolescents in Ndirande only. Future studies should consider increasing sample size and including both in and out of school adolescents. Secondly, data about participants’ socio-economic conditions and parental alcohol use were not collected. These details could have helped in exploring adolescent perspectives as affected by economic status and parental modelling.

## Conclusions

Adolescents view the ban on the sale of alcohol sachets in Malawi as a step towards reducing adolescent alcohol intake. However, quick to express that the recent packaging of the same alcohol presents challenges towards adolescents’ abstinence. Further, they emphasized that drinking or abstaining from alcohol is a choice one has to make as an individual. They also recommended policy enforcement on alcohol manufacturing and selling.

In light of our findings that cheaper and readily available alcohol is associated with increased underage use, greater considerations should be given to factors that regulate accessibility among adolescents. This includes enforcing minimum age limit and restricting sales to licensed places only. Considering the limitations in enforcement of alcohol policies described in this study, we advocate for intensified effort towards policy enforcement as well as parental involvement through different means such as positive role modelling and supervision of adolescents. We advocate efforts that aim to enlighten parents on the effects of social modelling of alcohol and parental alcohol use on adolescent drinking. Further, we recommend intensified efforts towards developing character traits and skills training to empower adolescents to delay initiation of alcohol use and counteract peer pressure.

## Data Availability

The datasets used are available from the corresponding author on request. Ethics approval and consent to participate. We obtained ethical approval from Malawi’s College of Medicine Research and Ethics Committee (COMREC- Number P.01/16/2071) prior to commencing the study. We obtained written informed consent from each participant prior to any study procedures. All participants were literate and provided verbal consent. We obtained a waiver from COMREC to obtain verbal consent. Participants were assured that their participation in the study will not affect their education at the schools. We maintained participants’ confidentiality, anonymity and privacy by holding interviews in a private and quiet room at the facility after working hours and weekends as per participant’s preference. We used codes instead of participants’ names in the study summaries. All audios and transcripts were saved in a password protected computer with limited access to the researchers.

## Abbreviations

COMREC: College of Medicine Ethics Committee
WHO: World Health Organization
IDIs: In-depth interviews
FGDs: Focus group discussions
MoEST: Ministry of Education, Science and Technology, Government of the Republic of Malawi
NIAAA: National Institute on Alcohol Abuse and Alcoholism
